# Producing Freestanding Single-Crystal BaTiO_3_ Films through Full-Solution Deposition

**DOI:** 10.3390/nano14171456

**Published:** 2024-09-07

**Authors:** Guoqiang Xi, Hangren Li, Dongfei Lu, Xudong Liu, Xiuqiao Liu, Jie Tu, Qianqian Yang, Jianjun Tian, Linxing Zhang

**Affiliations:** Institute for Advanced Materials Technology, University of Science and Technology Beijing, Beijing 100083, China

**Keywords:** perovskite oxides, ferroelectricity, freestanding thin film, full-solution deposition

## Abstract

Strontium aluminate, with suitable lattice parameters and environmentally friendly water solubility, has been strongly sought for use as a sacrificial layer in the preparation of freestanding perovskite oxide thin films in recent years. However, due to this material’s inherent water solubility, the methods used for the preparation of epitaxial films have mainly been limited to high-vacuum techniques, which greatly limits these films’ development. In this study, we prepared freestanding single-crystal perovskite oxide thin films on strontium aluminate using a simple, easy-to-develop, and low-cost chemical full-solution deposition technique. We demonstrate that a reasonable choice of solvent molecules can effectively reduce the damage to the strontium aluminate layer, allowing successful epitaxy of perovskite oxide thin films, such as 2-methoxyethanol and acetic acid. Molecular dynamics simulations further demonstrated that this is because of their stronger adsorption capacity on the strontium aluminate surface, which enables them to form an effective protective layer to inhibit the hydration reaction of strontium aluminate. Moreover, the freestanding film can still maintain stable ferroelectricity after release from the substrate, which provides an idea for the development of single-crystal perovskite oxide films and creates an opportunity for their development in the field of flexible electronic devices.

## 1. Introduction

Perovskite structures of transition-metal oxides have emerged as a large material system that provides a rich knowledge base for research in chemistry, physics, and materials science. The emergence of unique properties such as ferroelectricity/antiferroelectricity, ferromagnetism/antiferromagnetism, pyroelectricity, piezoelectricity, and superconductivity has led to a wide range of applications in the fields of spin-electronic devices, memristor devices, self-charging devices, energy storage, and photovoltaics [1,2,3,4,5]. At the same time, the demand for miniaturization and integration is becoming more urgent with the development of society. Therefore, the preparation of low-dimensional and ferroelectric oxide thin films has received much more attention and become one of the frontiers and hotspots in the study of ferroelectric materials. To date, various methods, including pulse laser deposition (PLD), radio-frequency (RF) magnetron sputtering, molecular beam epitaxy (MBE), and sol–gel/chemical-solution deposition, have been used to produce ferroelectric oxide films [6,7,8,9]. Based on this, various novel physical phenomena have been realized in epitaxial films via lattice mismatch, chemical substitution engineering, and interphase strain strategies [10,11,12]. Nevertheless, the application of these novelties and excellent performance capacities has been limited by the constraining effects of the substrate. More importantly, in this era in which flexible electronics shine, determining how to negate the limitations of conventional rigid substrates and make the most of the unique advantages of these films will be an issue we have to focus on.

Recently, a water-soluble strontium aluminate (Sr_3_Al_2_O_6_, SAO) sacrificial layer, applied to many perovskites and their heterostructures, has been reported, attracting unprecedented attention and leading to the development of freestanding perovskite oxide thin films [6,13,14,15]. Compared to conventional acid-etching, the laser lift-off process, inductively coupled plasma-reactive ion etching, and other ways of preparing self-supported thin films [16,17,18,19,20], the use of this layer has the advantages of being environmentally friendly and having a wide range of applications. In addition, perovskite oxide thin films with independence, transferability, and high-quality single-crystal thin film states can be achieved by using a water-soluble SAO sacrificial layer. For example, freestanding single-crystal ferroelectric barium titanate (BaTiO_3_, BTO) thin films with super elasticity and ultraflexibility were synthesized using PLD in the presence of an SAO sacrificial layer [21], which exhibited stress-activated modulation of electric dipole rotation. Huang et al. reported that sub-one-nanometer transferrable ultrahigh-*κ* single-crystal strontium titanate (SrTiO_3_, STO) films integrated with two-dimensional layered semiconductors effectively reduced capacitance equivalent thickness and thus allowed the obtainment of high-quality dielectric–channel interfaces [22]. Additionally, based on the SAO water-soluble layer, researchers have conceived of the idea of transferring high-density switchable skyrmion-like polar nanodomains onto silicon, which is important for the application of the rich physical properties of single-crystal oxides [23]. Despite the many reports on the realization of freestanding perovskite oxides thin films based on the use of SAO sacrificial layers, it is still challenging to reduce the preparation threshold and simplify the preparation methods as these films are mainly synthesized based on high-vacuum techniques, such as reactive MBE and PLD techniques [24,25]. In order to develop a cost-effective method, Salles et al. obtained freestanding CoFe_2_O_4_ membranes by combining atomic layer deposition and solution deposition processing using a sacrificial layer of SAO [26], providing a good idea for further research on the development of self-supported oxide films [27].

In this study, we investigated the preparation of freestanding single-crystal BTO thin films using a simple, easy-to-develop, and low-cost chemical full-solution deposition method. Molecular dynamics (MD) simulations provided effective evidence useful for the realization of full-solution deposition. They suggested that solvent molecules with high binding energies preferentially absorb on the SAO surface to form an effective protective layer and inhibit the hydration reaction, which in turn enabled us to prepare freestanding perovskite oxide single-crystal films on the SAO sacrificial layer using full-solution deposition. We also demonstrated that the freestanding BTO film released from the substrate retains its properties without significant changes, providing an idea for the preparation of freestanding perovskite oxide thin films.

## 2. Materials and Methods

Strontium acetate (99%, Aladdin, Shanghai, China), barium acetate (98%, Aladdin), aluminum acetylacetonate (99%, Aladdin), titanium isopropoxide (TTIP, Aldrich, 97%), acetic acid (99.5%, Sinopharm Chemical, Shanghai, China), and 2-methoxyethanol (99%, Aladdin) were purchased and directly used without any purification. The SAO precursor solution with a concentration of 0.25 mol L^−1^ was prepared as follows. 0.1028 g strontium acetate and 0.1621 g aluminum acetylacetonate were dissolved in 2 mL acetic acid and stirred for two hours at 120 °C until the solution was completely clear. The BTO precursor solution also was prepared at a concentration of 0.2 mol L^−1^, 0.2554 g barium acetate was dissolved in 573 μL acetic acid and stirred for two hours at 120 °C until the solution was completely clear. Then 305 μL TTIP and 2455 μL 2-methoxyethanol were added to obtain BTO precursor solution. In this process, 2-methoxyethanol was used as a solvent, acetic acid was used as a chelating agent, and other metal salts were used as raw materials. In the solution, the addition of acetic acid leads to a dehydration condensation reaction and effectively promotes the formation of a network-like structure of metal ions. It is worth noting that the BTO precursor solution need to be prepared in an atmosphere of N_2_. The SAO and BTO solutions were filtered with a PTFE filter with a 0.22 μm pore size. Subsequently, the epitaxial SAO thin films were fabricated on STO (001) substrate via spin-coating technique at 5000 rpm for 30 s. The wet films were then heated at 150 °C and 230 °C for 5 min, respectively. Thereafter, the samples were transferred to a muffle furnace and annealed at 850 °C for 30 min to obtain epitaxial SAO thin films. Furthermore, the BTO thin films were fabricated on SAO/STO using a similar technique. The BTO solution was deposited at 3000 rpm for 30 s, and the wet films were pyrolyzed at 300 °C for 10 min to decompose the organics in the films. Finally, the samples were annealed at a temperature of 850 °C with a heating rate of 5 °C min^−1^.

The process of releasing the freestanding films is described as follows: The BTO/SAO/STO heterostructure surface adhered onto the flexible protective layer (PDMS or PET) surface. Subsequently, it was immersed in deionized water to remove the SAO layer. The BTO film was released on the flexible substrate after being immersed for more than one day.

The crystal structures of the films were investigated using X-ray diffraction (XRD, PANalytical X’Pert Powder PW3040/60, Almelo, The Netherlands) with Cu Kα radiation (1.54056 Å). Reciprocal space mapping (RSM) measurements were carried out at the diffuse X-ray scattering station of the Beijing Synchrotron Facility (1W1A beamline). The surface morphologies and thicknesses of films were characterized via scanning electron microscopy (SEM, SU4800, Hitachi, Tokyo, Japan). Piezoresponse force microscopy (PFM) images were taken using an environmental chamber equipped with Agilent 5500AFM/SPM (Agilent Technologies, Santa Clara, CA, USA).

The nature of the interaction between the SAO layer and BTO precursor was ascertained by using the molecular mechanics method as implemented in the Forcite module of the Materials Studio 2019 simulation software. The crystal model of SAO was composed of 2 × 2 × 1 unit cells. The modeling thickness of the crystal was 15.9 Å. We prepared the crystal structure such that there were four different terminal cutoff surfaces along the *z* direction. Individual solvent molecules of 2-methoxyethanol, acetic acid, and water were built above the SAO substrates, and the thickness of the vacuum layer was 30 Å. In the simulations, the condensed-phase optimized molecular potentials for an atomistic simulation studies (COMPASS-II) forcefield was used to optimize the structures of interest. In geometry optimization, all systems were allowed to relax until the energy, maximum force, and displacement were less than 2 × 10^−5^ Ha, 0.001 Ha/Å, and 1 × 10^−5^ Å, respectively. The adsorption energy (*E*_a_) was calculated as follows: *E*_a_ = *E*_t_ − (*E*_s_ + *E*_m_), where *E*_t_, *E*_s_, and *E*_m_ represent the total energy of the SAO surface together with the solvent molecules, clean SAO surface, and solvent molecules, respectively. The binding energy (*E*_b_) between the solvent molecules and the SAO surface is expressed as *E*_b_ = −*E*_a_.

## 3. Results and Discussion

Sr_3_Al_2_O_6_ has a similar structure to tricalcium aluminate (Ca_3_Al_2_O_6_), which is one of the main phases of normal Portland cement clinker and an important component with respect to cement’s setting and hardening behavior [28]. The introduction of strontium instead of calcium significantly increases this compound’s solubility in water, which is one of the main advantages of its use as a sacrificial layer. The crystal structure of SAO is cubic (space group *Pa*$\bar{3}$) with lattice dimensions of *a* = 15.844 Å (Figure S1a). It can easily be found that the lattice structure of SAO (Figure S1b) is extremely similar to our common epitaxial substrates, such as STO and LaAlO_3_ (LAO). STO is an idealized cubic structure (with the space group *Pm*$\bar{3}$*m*) with lattice parameters of *a* = 3.905 Å at room temperature [29], while LAO is a rhombohedral distorted perovskite structure (with the space group *R*$\bar{3}$*c*) that can also be described as psuedocubic, with lattice parameters of 3.791 Å [30]. The lattice mismatch between LAO and SAO is much larger than that between SAO and STO. The lattice parameter of STO is about one-fourth of that for SAO, making it more suitable for the epitaxial growth of SAO. Encouragingly, this also makes it very feasible to prepare epitaxial SAO/perovskite oxide heterostructures, such as BaTiO_3_, PbTiO_3_, BiFeO_3_, etc. [14,31,32,33]. Therefore, the preparation of freestanding perovskite oxide films via the lift-off technique, utilizing the advantage of the extreme water solubility of SAO, has received unprecedented attention and development.

**Figure 1:**
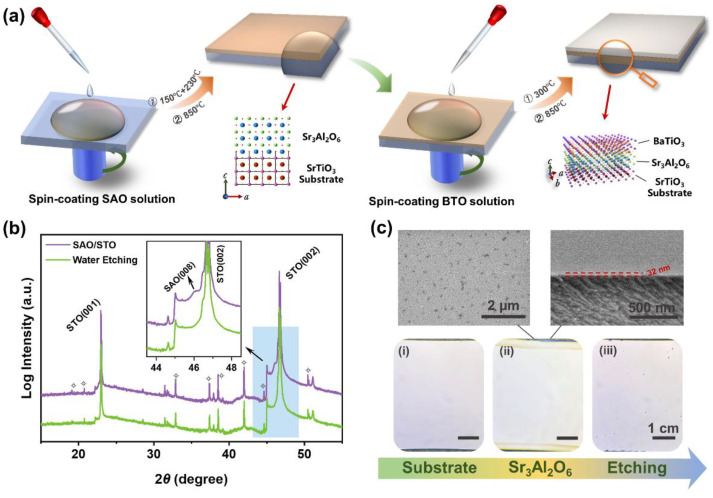
Schematic of the preparation of BaTiO_3_/Sr_3_Al_2_O_6_/SrTiO_3_ heterogeneous structures via chemical solution deposition (**a**). X-ray diffraction pattern of Sr_3_Al_2_O_6_ before and after water etching (**b**). ‘✧’ represents the diffraction peak of the substrate. The surface optical pictures (bottom) of pure SrTiO_3_ (**i**), Sr_3_Al_2_O_6_/SrTiO_3_ (**ii**), and after water etching (**iii**), and the surface morphology and cross-section image (top) of the Sr_3_Al_2_O_6_ thin film (**c**).

To realize preparation of freestanding thin films via the full-solution deposition method, we tried using spin-coating deposition. Figure 1a provides a schematic of the process of spin-coating preparation. The SAO precursor solution was uniformly spread on the STO (001) substrate via a spin-coating process, followed by two steps of pyrolysis and annealing to obtain the epitaxial SAO thin film. The successful growth of epitaxial films was verified using the XRD pattern, as shown in Figure 1b. The XRD pattern clearly shows the SAO (008) diffraction peaks, and no diffraction peaks of other phases are observable, indicating the high phase purity of the films. ‘✧’ represents the X-ray diffraction peaks of the SrTiO_3_ substrate under Cu K_β_, W L_α_, W L_β_, and W L_γ_ (Figure S2). In addition, the surface morphology and cross-section image of the SAO thin film were observed, as shown in Figure 1c. Although there were some holes on the surface of the film, the grain size of the film was still relatively uniform and showed good crystallinity, providing excellent conditions for the preparation of heterogeneous structures. Furthermore, we verified the solubility of the SAO thin films in water. Macroscopically, optical pictures of pure STO, SAO/STO, and water-etched surfaces were compared (Figure 1c). By comparing the optical images of pure STO, SAO/STO and water-etched surfaces on a macroscopic scale, we can observe the degradation of SAO films in water more intuitively. Moreover, the XRD pattern also shows that the diffraction peaks of SAO completely disappear after water etching, indicating that we have successfully prepared water-soluble epitaxial SAO thin films on an STO (001) substrate via the spin-coating method.

**Figure 2:**
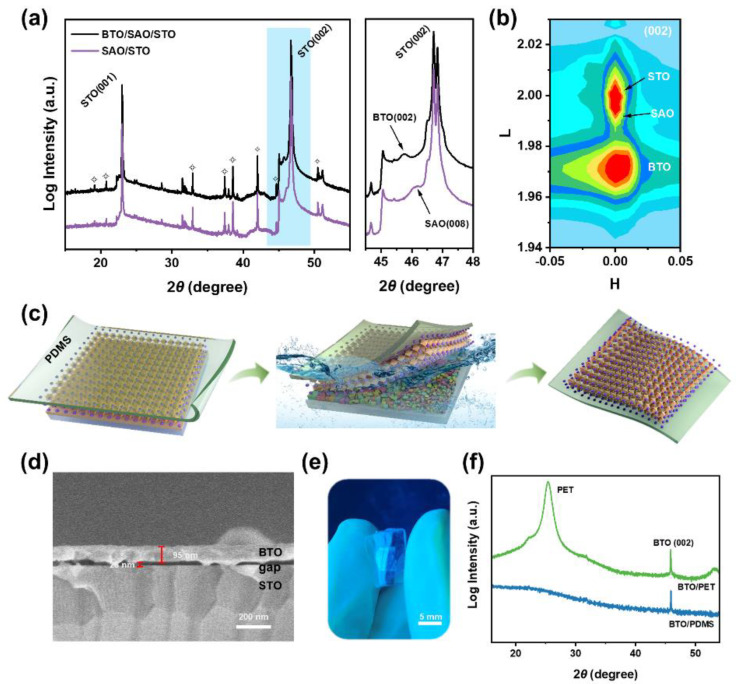
X-ray diffraction pattern (**a**) and synchrotron X-ray reciprocal space mappings (**b**) of BaTiO_3_/Sr_3_Al_2_O_6_/SrTiO_3_ heterogeneous structures. A schematic of the release process of BaTiO_3_ films from the substrate (**c**). Cross-sectional SEM image of the Sr_3_Al_2_O_6_ layer in the middle of the heterostructure after one day of immersion in deionized water (**d**). Optical microscopy images of freestanding BaTiO_3_ film (5 × 5 mm) released on polydimethylsiloxane (**e**). X-ray diffraction pattern of the freestanding BaTiO_3_ films on the polydimethylsiloxane and polyethylene terephthalate flexible layer (**f**).

However, the major challenge in this work was determining how to successfully grow perovskite oxide films epitaxially on SAO/STO structures using chemical solution deposition (spin-coating), implying that the preparation of the precursor solution would play an important role in the whole process. To avoid damaging the SAO sacrificial layer during the deposition of the perovskite oxide film, we chose to use 2-methoxyethanol and acetic acid as solvents for the preparation of the precursor. In addition, BTO-based materials with excellent ferroelectric and piezoelectric properties were selected for the epitaxial preparation of freestanding films [34,35]. Experimentally, a certain amount of barium acetate was dissolved in acetic acid, and the resulting solution was subsequently mixed with a solution of 2-methoxyethanol containing titanium isopropoxide to obtain the BTO precursor solution. As with the preparation of SAO films, spin-coating deposition was used to spread the films on the SAO/STO structure, and then pyrolysis and annealing processes were induced to obtain BTO single-crystal films. Excitingly, the XRD patterns (Figure 2a) showed that the BTO film was successfully epitaxially grown on the SAO/STO structure through full-solution deposition. A second phase did not appear in the XRD pattern, demonstrating high phase purity. Further observation of the XRD patterns indicates that following the epitaxial growth of the BTO film (Figure S3), the diffraction peaks corresponding to SAO persist, albeit with reduced intensity. The reason for this may be that the redeposition of the BTO film caused some interference with the diffraction of SAO. Due to the coverage of BTO, the number of X-rays reaching the SAO layer is reduced during the out-of-plane theta-2theta linkage XRD scan, so the intensity of SAO may be affected. Of course, we cannot rule out that the decrease in SAO intensity may be related to a partial loss of the SAO layer or deterioration of crystal quality. Although the organic solvent can isolate the contact between water and SAO to a certain extent, it cannot completely negate the damage water inflicts on SAO. Fortunately, further growth and release of the BTO layer can still be achieved even if there is a partial loss of SAO. In addition, the results of the synchrotron X-ray reciprocal space mapping (RSM) conducted around the (002) reflection of the BTO/SAO/STO heterostructures were further analyzed (Figure 2b), with the findings confirming that epitaxial growth of BTO thin films on SAO/STO structures can indeed be achieved through chemical deposition methods.

Subsequently, the BTO/SAO/STO heterostructures were immersed in deionized water to etch off the SAO layer to release the BTO film from the heterostructure (Figure 2c). It can be observed in Figure 2d that the SAO layer in the middle of the sandwich structure disappeared from the cross-section SEM image after being immersed in deionized water for one day, revealing an obvious gap. The appearance of this phenomenon laid a strong foundation for us to realize the lift-off of the BTO epitaxial films. Moreover, the cross-sectional SEM image also shows that the thickness of the BTO film is approximately 95 nm. To facilitate the transfer of the film after release, a flexible support layer needed to be attached to the surface of the heterostructure before immersion. The optical microscopy images of freestanding BTO film released on a polydimethylsiloxane (PDMS) support layer are shown in Figure 2e. It shows that the freestanding BTO film has a distinct crystalline luster and an almost complete morphology and size (5 × 5 mm). Furthermore, the (002) diffraction peak of BTO could be detected from the XRD pattern (Figure 2f) after releasing the freestanding BTO films on the PDMS and polyethylene terephthalate (PET) flexible substrates, respectively, indicating that the BTO layer still retained its epitaxial structure and confirming the feasibility of the full-solution deposition strategy for the preparation of freestanding films. Also, it can be noticed that the shape of the diffraction peak of the BTO changes from broad to thin. In our opinion, the shape of the diffraction peaks may be affected by various factors. From the perspective of XRD measurements, the test device used before and after peel-off should be the same and have a defined wavelength, which, ideally, should maintain the same shape of the diffraction peaks. However, during the course of an experiment, several uncertainties can affect the shapes of diffraction peaks despite efforts to maintain consistency in experimental conditions. For example, the process of etching the SAO sacrificial layer involves immersion, which can introduce small structural changes due to the diffusion of trace elements like Ba within the film. This diffusion can alter the crystal structure or composition slightly, impacting the shapes of diffraction peaks observed during XRD analysis. In addition, the surface morphology of the BTO films exposed before and after the transfer can also change due to the transfer process, potentially resulting in alterations of the shapes of the diffraction peaks. Moreover, the release of substrate clamping stresses and bending forces during the transfer process may also induce subtle changes in the lattice at the surface and/or interface. Their combined effect may equally have been the cause of the decrease in the intensity of the (001) diffraction peak of the freestanding BTO film. Therefore, responsibility for making the diffraction peak shapes not completely consistent can be attributed to a combination of influencing factors, and the best judgement is still not available with regard to our current technique, which will encourage us to further explore this issue in future research. Moreover, due to the release of partial clamping stresses, the diffraction peaks of the released BTO films appear at a higher angle, a finding that is in agreement with previous reports [32,36] and indicates the feasibility of the full-solution method for preparing freestanding films.

**Figure 3:**
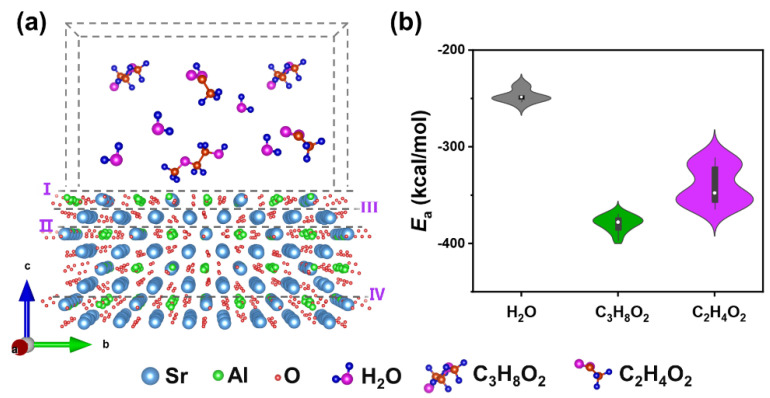
Molecular simulation modeling of Sr_3_Al_2_O_6_ surfaces and solvents (**a**). Statistical diagram of molecular adsorption energy of different solvents (**b**).

In order to obtain strong supporting evidence with which to confirm the feasibility of the full-solution method, we used MD simulations to investigate the surface adsorption behavior of the main solvent molecules in the BTO precursor on SAO. MD simulations have been widely applied to probe the adsorption behavior at liquid–solid, gas–solid, and liquid–gas interfaces [37,38,39,40,41]. It is not only a powerful means with which to study adsorption behavior at the microscopic molecular level but also the intrinsic reason for preparing freestanding films via the full-solution method, as results regarding this behavior can easily be obtained with the help of MD simulations. In this study, the SAO (00*l*) surface was selected as the adsorption substrate since the SAO thin films were epitaxially grown on (001)-oriented STO. The crystal model of SAO was composed of 2 × 2 × 1 unit cells. The modeling thickness of the crystal was 15.9 Å, and the thickness of the vacuum layer was 30 Å. During the simulating procedure, the sizes of the simulation supercells in the *a*, *b*, and *c* directions were constant, while the properties of the upper-terminal surfaces (Figure S4) exposed to a vacuum were obtained by cutting the crystal models. Considering the crystal structure of SAO, we cut the super cells to obtain four different terminal cutoff surfaces and labelled them as I, II, III, and IV, respectively (Figure 3a); the matched top views are shown in Figure S4. The difference between each cutoff surface lies in the varying arrangement of exposed atoms, which results in different adsorption capacities for solvent molecules and facilitates the analysis of the intrinsic mechanism of freestanding thin films prepared via the full-solution method. Regarding the solvent molecules, 2-methoxyethanol and acetic acid, which are the main solvent molecules in the chemical deposition process [42,43], were chosen for comparison with water in MD simulations. The adsorption energy (*E*_a_) [44] was calculated as follows: *E*_a_ = *E*_t_ − (*E*_s_ + *E*_m_), where *E*_t_, *E*_s_, and *E*_m_ represent the total energy of the SAO surface together with the solvent molecules, clean SAO surface, and solvent molecules, respectively. The binding energy (*E*_b_) between the solvent molecules with the SAO surface is expressed as *E*_b_= −*E*_a_. As shown in Figure S5, the adsorption sites and adsorption energies of each of the three molecules on four different terminal surfaces are presented, where the black, magenta, and green values represent the adsorption energies of water, acetic acid, and 2-methoxyethanol, respectively. The *E*_a_ of water is about −250 kcal/mol, while that of acetic acid and 2-methoxyethanol ether is below −300 kcal/mol. It was not hard to determine that the *E*_a_ of 2-methoxyethanol and acetic acid molecules is much smaller than that of water (Figure 3b). Inversely, smaller adsorption energies indicate higher binding energies, indicating that 2-methoxyethanol and acetic acid not only have higher binding energies with respect to the SAO surface but are also more likely to preferentially bind to the surface. In essence, these molecules combine with the lone-pair electrons of water principally via coordination when SAO reacts with water. The Sr–O bonds located at the centers of the six AlO_4_ tetrahedra (Figure S1c) easily react with water and break, thus collapsing the crystal structure and generating hydration products [13,45]. It is reasonable to speculate that when the three solvent molecules are included in the same solution system, either 2-methoxyethanol or acetic acid will be preferentially adsorbed on the surface of the SAO film to form a protective layer. Therefore, the presence of the protective layer is significant in that it helps to inhibit the reaction of water molecules with the SAO, generating suitable conditions for realizing the feasibility of preparing epitaxial thin films on SAO films. Critically, the MD simulation also provides an important reference for realizing the preparation of freestanding oxide perovskite structure films using the strategy of chemical full-solution deposition.

**Figure 4:**
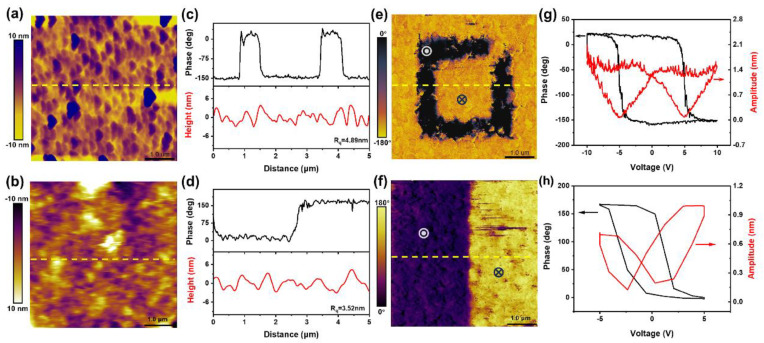
Atomic force microscopy images of the surface morphologies of BaTiO_3_ film before (**a**) and after the release (**b**). The red and black line profiles indicate the surface morphologies and the piezoresponse force microscopy (PFM) phases of the BaTiO_3_ film before and after the release (**c**,**d**), respectively. PFM phase images of BaTiO_3_ film before (**e**) and after the release (**f**), which were generated by scanning the film surface with a tip under ±10 V bias (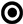 and 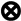 represent +10 V and −10 V, respectively). Local PFM amplitude (red) and phase (black) hysteresis curves were acquired on BaTiO_3_ films before (**g**) and after the release (**h**).

Considering the stability of the quality and properties of the BTO film after it was peeled from the substrate, we first investigated the morphological changes of the films before and after release using atomic force microscopy (AFM). The surface morphologies before and after the release are shown in Figure 4a and Figure 4b, respectively. It can be observed that there is variability in their morphology, which may be due to the fact that the front and back sides of the film were reversed with respect to each other after the film had been released on the flexible substrate. The red curves in Figure 4c,d indicate the surface height undulations along the yellow dashed line down in Figure 4a and Figure 4b, respectively. Furthermore, the root-mean-square roughness values of the BTO films before and after the release are 4.89 and 3.52 nm, respectively. Therefore, this change in root-mean-square roughness could also be caused by the reversal of the front and back surfaces of the film during release. Furthermore, the ferroelectric properties of the BTO films were probed through piezoresponse force microscopy (PFM) using conductive AFM tip scanning across a 5 μm × 5 μm area. The out-of-plane PFM phase signals of the original and post-release films at ±10 V polarization are shown in Figure 4e and Figure 4f, respectively. The black curves in Figure 4c,d represent the phase signals along the yellow dashed line down in Figure 4e and Figure 4f, respectively, which suggest a significant phase inversion between regions previously exposed to positive or negative bias. Crucially, this finding suggests that opposite polarization states exist between the two regions and can be switched via an applied voltage. The local hysteresis loops in phase and amplitude are also evidence of freestanding BTO film ferroelectricity, a finding that is consistent with the pre-transfer film having a phase flip of nearly 180° and a butterfly amplitude curve (Figure 4g,h). At the same time, the freestanding BTO film still exhibited polarization switching in response to the electric field under cyclic polarizations (Figure S6), indicating that the ferroelectricity of the BTO films was maintained after release from the substrate. The domain walls can still be observed after ten minutes in the phase and amplitude images, further indicating that the film is ferroelectric (Figure S7). Therefore, by combining all the analysis results, we can conclude that the preparation of freestanding perovskite oxide single-crystal thin films via full-solution deposition is technically feasible. The successful application of this method will provide unprecedented opportunities for the development of low-dimensional materials.

## 4. Conclusions

In summary, we have proposed a strategy for the preparation of perovskite oxide thin films on water-soluble sacrificial layers using a cost-effective and easily scalable full-solution deposition method. The strategy was theoretically supported by molecular dynamics simulations and also subjected to practical validation. Freestanding BTO films were prepared using this strategy to maintain stable ferroelectricity after transfer, and these films exhibited no significant differences compared to those prepared using other physical methods. Excitingly, we expect that this method will also be applied to other perovskite oxide materials, broadening the way for the exploration of their novel physical properties as well as applications in low-dimensional electronic devices.

## Data Availability

All data used in this study are available upon request from the corresponding author.

## Supplementary Materials

The following supporting information can be downloaded at: https://www.mdpi.com/article/10.3390/nano14171456/s1, Figure S1. The crystal structure of Sr_3_Al_2_O_6_ in different views, where (b) has two unit cells along the *b* direction; Figure S2. X-ray diffraction pattern of Sr_3_Al_2_O_6_ before and after water etching as well as pure SrTiO_3_ substrates. ‘✧’ represents the X-ray diffraction peaks of the SrTiO_3_ substrate under Cu K_β_, W L_α_, W L_β_, and W L_γ_, respectively; Figure S3. Enlarged patterns of SrTiO_3_ substrate, Sr_3_Al_2_O_6_/SrTiO_3_ films and BaTiO_3_/Sr_3_Al_2_O_6_/SrTiO_3_ films around the (001) (a) and (002) (b) diffraction peaks of the SrTiO_3_ substrate; Figure S4. Four different terminal cutoff surfaces and labeled them as I (a), II (b), III (c), and IV (d), respectively; Figure S5. Adsorption positions and energies of solvent molecules on terminal cutoff surfaces of I (a), II (b), III (c), and IV (d), respectively. The black, magenta, and green values represent the adsorption energies of water, acetic acid, and 2-methoxyethanol on the surface, respectively; Figure S6. Local phase and amplitude hysteresis loops of piezoelectric response for the freestanding BaTiO_3_ film, demonstrating the ferroelectric switching; Figure S7. The PFM images dependence on the delay time of freestanding BaTiO_3_ film switched at ±10 V DC voltage (,  represent +10 V and −10 V, respectively). The domain walls can still be observed in the phase and amplitude images after 10 min, indicating that the film is ferroelectric. There is some attenuation of the signal, probably due to incomplete polarization switching in the film by the low polarization voltage (The maximum polarization voltage for PFM instruments is ±10 V). And the presence of a built-in electric field in the transferred film also leads to the depolarization phenomenon and produces a negative effect.
