# Supplementary material for: Producing Freestanding Single-Crystal BaTiO3 Films through Full-Solution Deposition
Source: Nanomaterials (Basel). 2024 Sep 7;14(17):1456. doi: 10.3390/nano14171456 (PMC11396833; doi:10.3390/nano14171456)
Supplement: Supplementary file 1 [file nanomaterials-14-01456-s001.zip › nanomaterials-3182699-supplementary.pdf]

Supplementary Materials for

Producing Freestanding Single-crystal $BaTiO_3$ Films through Full-Solution Deposition

Guoqiang Xi [†], Hangren Li [†], Dongfei Lu, Xudong Liu, Xiuqiao Liu, Jie Tu, Qianqian Yang, Jianjun Tian, Linxing Zhang *

Institute for Advanced Materials Technology, University of Science and Technology Beijing, Beijing 100083, China; b20200657@xs.ustb.edu.cn (G.X.); d202110713@xs.ustb.edu.cn (H.L.); m202121385@xs.ustb.edu.cn (D.L.); m202111359@xs.ustb.edu.cn (X.L.); m202121379@xs.ustb.edu.cn (X.L.); g20209405@xs.ustb.edu.cn (J.T.); d202210745@xs.ustb.edu.cn (Q.Y.); tianjianjun@mater.ustb.edu.cn (J.T.)

* Correspondence to: linxingzhang@ustb.edu.cn;

[†] These authors have the same contribution to the work.

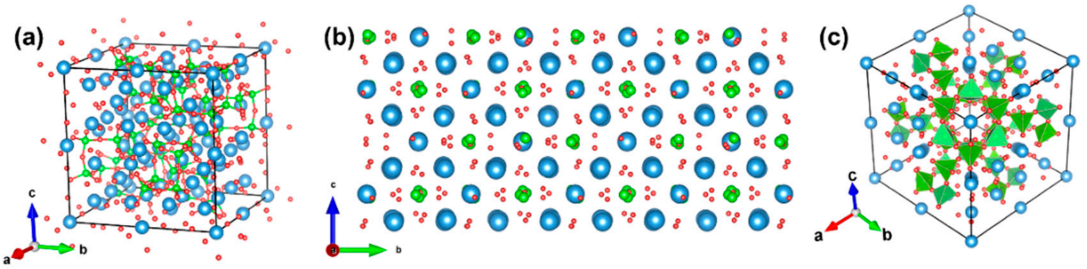

Figure S1. The crystal structure of $Sr_3Al_2O_6$ in different views, where (b) has two unit cells along the *b* direction.

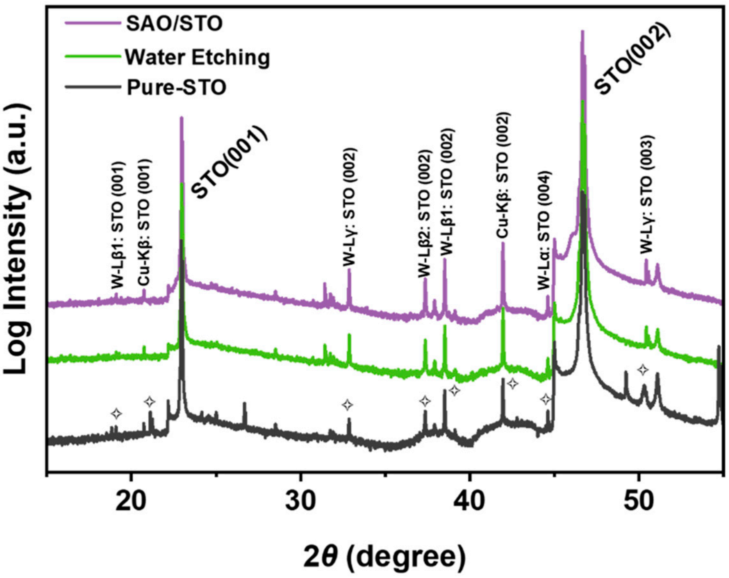

Figure S2. X-ray diffraction pattern of $Sr_3Al_2O_6$ before and after water etching as well as pure $SrTiO_3$ substrates. '✧' represents the X-ray diffraction peaks of the $SrTiO_3$ substrate under Cu K_β, W L_α, W L_β, and W L_γ, respectively.

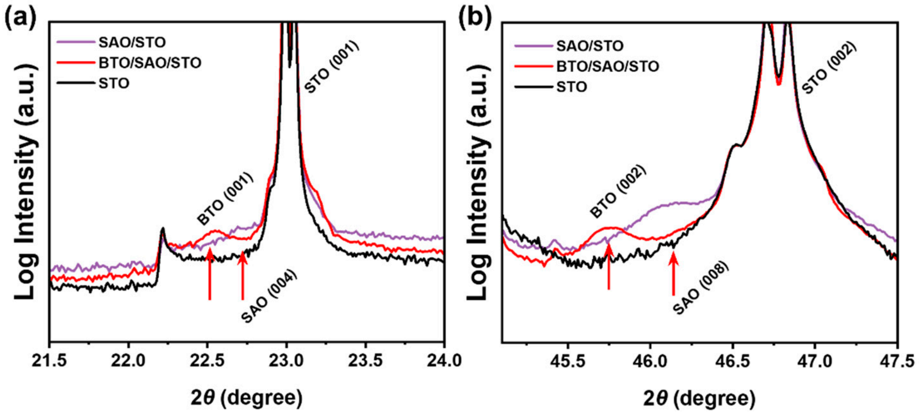

Figure S3. Enlarged patterns of $SrTiO_3$ substrate, $Sr_3Al_2O_6/SrTiO_3$ films and $BaTiO_3/Sr_3Al_2O_6/SrTiO_3$ films around the (001) (a) and (002) (b) diffraction peaks of the $SrTiO_3$ substrate.

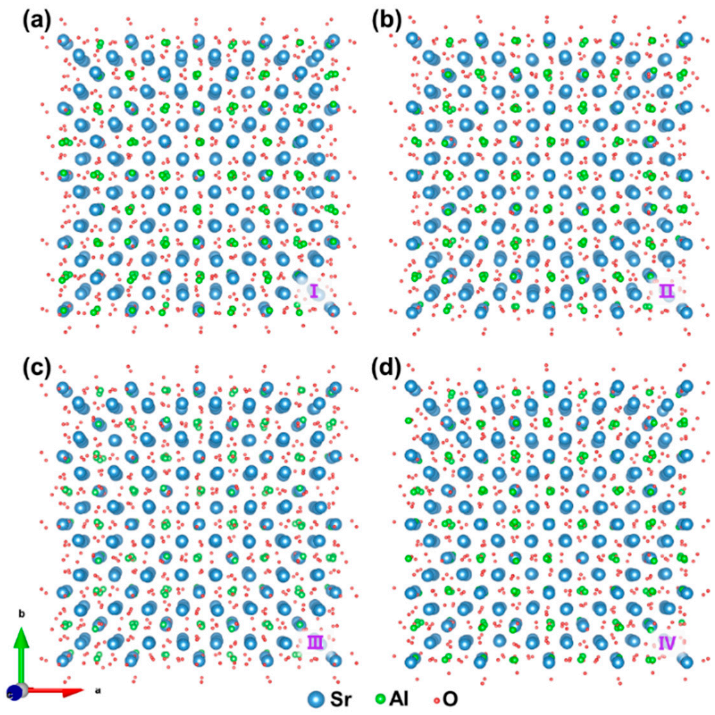

Figure S4. Four different terminal cutoff surfaces and labeled them as I (a), II (b), III (c), and IV (d), respectively.

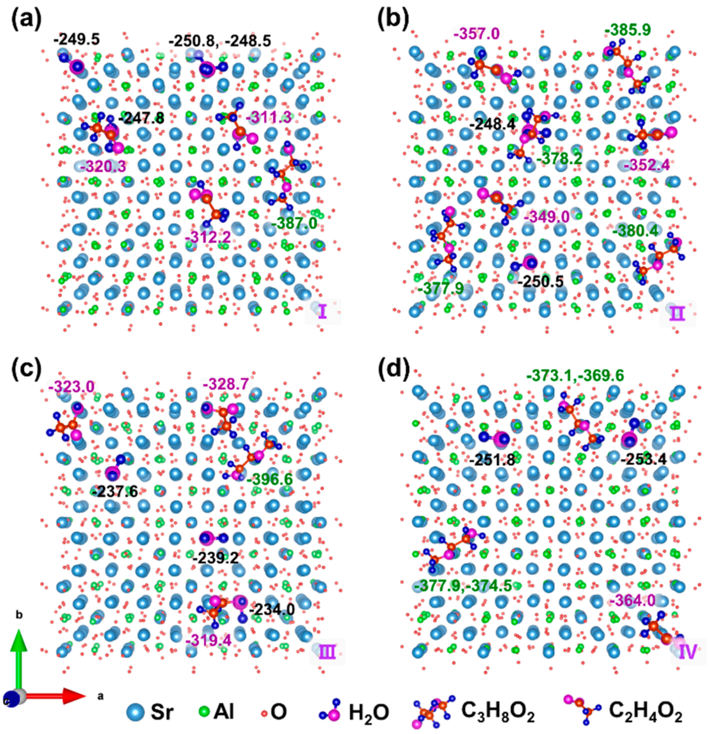

Figure S5. Adsorption positions and energies of solvent molecules on terminal cutoff surfaces of I (a), II (b), III (c), and IV (d), respectively. The black, magenta, and green values represent the adsorption energies of water, acetic acid, and 2-methoxyethanol on the surface, respectively.

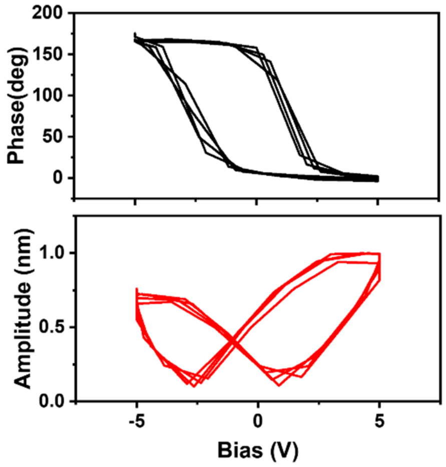

Figure S6. Local phase and amplitude hysteresis loops of piezoelectric response for the freestanding $BaTiO_3$ film, demonstrating the ferroelectric switching.

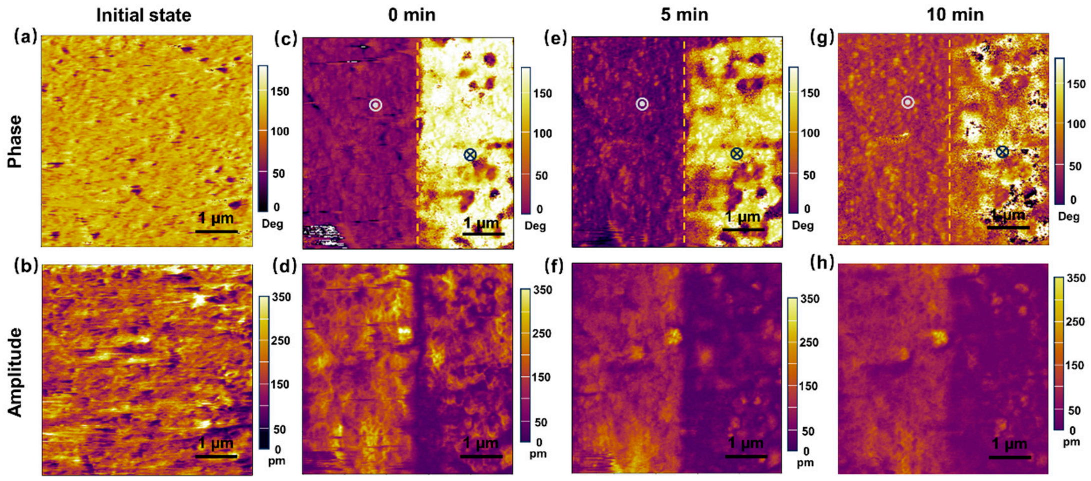

Figure S7. The PFM images dependence on the delay time of freestanding $BaTiO_3$ film switched at ±10V DC voltage (⊙, ⊗ represent +10V and -10V, respectively.). The domain walls can still be observed in the phase and amplitude images after 10 minutes, indicating that the film is ferroelectric. There is some attenuation of the signal, probably due to incomplete polarization switching in the film by the low polarization voltage (The maximum polarization voltage for PFM instruments is ±10V). And the presence of a built-in electric field in the transferred film also leads to the depolarization phenomenon and produces a negative effect.
